# Overshoot of the Respiratory Exchange Ratio during Recovery from Maximal Exercise Testing in Kidney Transplant Recipients

**DOI:** 10.3390/ijerph18179236

**Published:** 2021-09-01

**Authors:** Alessandro Patti, Daniel Neunhaeuserer, Andrea Gasperetti, Veronica Baioccato, Marco Vecchiato, Francesca Battista, Francesco Marchini, Marco Bergamin, Lucrezia Furian, Andrea Ermolao

**Affiliations:** 1 Sport and Exercise Medicine Division, Department of Medicine, University Hospital of Padova, Via Giustiniani, 2, 35128 Padova, Italy; alessandropatti88@gmail.com (A.P.); a.gasperetti@libero.it (A.G.); veronicabaioccato@gmail.com (V.B.); marcovecchiato.md@gmail.com (M.V.); francesca.battista@phd.unipd.it (F.B.); marco.bergamin@unipd.it (M.B.); andrea.ermolao@unipd.it (A.E.); 2 Clinical Network of Sports and Exercise Medicine of the Veneto Region, 35128 Padova, Italy; 3 Department of Medicine, Nephrology, University Hospital of Padua, 35128 Padova, Italy; francescomarchini1454@gmail.com; 4 Kidney and Pancreas Transplantation Unit, University Hospital of Padua, 35128 Padua, Italy; lucrezia.furian@unipd.it

**Keywords:** cardiopulmonary exercise test, renal transplantation, exercise, nephrology, functional evaluation

## Abstract

The overshoot of the respiratory exchange ratio (RER) during recovery from exercise has been found to be reduced in magnitude among patients with heart failure. The aim of this study is to investigate whether this phenomenon could also be present in patients with peripheral, and not cardiac, limitations to exercise such as kidney transplant recipients (KTRs). In this retrospective cross-sectional study, KTRs were evaluated with maximal cardiopulmonary exercise testing (CPET) assessing the RER overshoot parameters during recovery: the RER at peak exercise, the maximum RER value reached during recovery, the magnitude of the RER overshoot (RER mag = (RER max-peak RER)/peak RER%) and the linear slope of the RER increase after the end of exercise. A total of 57 KTRs were included in the study (16 females), all of them showing a significant RER overshoot (RER mag: 28.4 ± 12.7%). Moreover, the RER mag showed significant correlations with the fitness of patients (peak VO_2_: ρ = 0.57, *p* < 0.01) and cardiorespiratory efficiency (VE/VCO_2_ slope: r = −0.32, *p* < 0.05; oxygen uptake efficiency slope (OUES): r = 0.48, *p* < 0.01). Indeed, the RER mag was significantly different between the subgroups stratified by Weber’s fitness class or a ventilatory efficiency class. Our study is the first to investigate recovery of the RER in a population of KTRs, which correlates well with known prognostic CPET markers of cardiorespiratory fitness, determining the RER mag as the most meaningful RER overshoot parameter. Thus, the RER recovery might be included in CPET evaluations to further improve prognostic risk stratifications in KTRs and other chronic diseases.

## 1. Introduction

Cardiopulmonary exercise testing (CPET) is currently recognized as a useful clinical tool to assess the physical fitness and exercise limitations of patients with cardiac, pulmonary and musculoskeletal diseases [1,2,3]. Its value has been demonstrated in the prevention and prognostic monitoring of cardiovascular diseases, in the prescription and follow-up of exercise training interventions and in the evaluation of medical treatment measures [1,4]. Indeed, several of the parameters determined during CPET, such as evaluating the physical fitness of patients (i.e., peak oxygen uptake: peak VO_2_) and cardiorespiratory efficiency (i.e., the ventilation to carbon dioxide production ratio: VE/VCO_2_) during exercise, have been shown to be well-consolidated prognostic markers in patients with heart failure. Moreover, a CPET evaluation has an important role in driving clinical decisions in end-stage heart or lung disease, guiding the selection process of candidates for transplantation [4]. However, the current body of evidence about CPET is mainly focused on the exercise response [2,3]; fewer studies have investigated the behavior of respiratory gas indices during recovery after maximal exercise testing especially in clinical populations.

At present, there seems to be a reasonable agreement on the fact that the recovery of VO_2_ after maximal exercise testing is delayed in patients with heart failure and that this delay correlates with the severity of the disease [5,6]. Moreover, the VO_2_ recovery delay has been correlated with a worse prognosis in more than one study [7,8]. The reasons underlying this phenomenon have not yet been completely elucidated; a few authors have attributed this delay to the repayment of the O_2_ deficit from exercise [7] whereas others have attributed it to the replenishment of muscle energy stores and especially the resynthesis of high-energy phosphates [5].

Another associated phenomenon that has been observed in patients with heart failure is the overshoot of VO_2_ at the end of exercise [9]. This is represented by a transitory increase in VO_2_ during the first part of recovery and has been attributed to a paradoxical increase in the stroke volume due to a reduction in systemic vascular resistances. Speculations have been made about whether this impaired relationship between the stroke volume and vascular resistances could be due to a slow decrease in the adrenergic tone or to the repayment of the accumulated O_2_ deficit [10,11].

Recently, attention has been drawn to the overshoot of several other CPET indices during recovery after maximal exercise. In particular, the overshoot of the respiratory exchange ratio (RER: VCO_2_/VO_2_), ventilation to oxygen consumption ratio (VE/VO_2_) and the end-tidal partial pressure of oxygen (PETO_2_) commonly observed in healthy subjects after maximal exercise testing were found to be reduced in magnitude in patients with cardiac disease (left ventricular ejection fraction (LVEF) < 40%). Takayanagi et al. demonstrated how the magnitude of the overshoot (i.e., the percentual increase during recovery compared with the peak value) of the aforementioned indices was lower in cardiac patients than in healthy subjects. The explanation for this phenomenon has been attributed to the delayed recovery of VO_2_ typically present in these patients. Moreover, the authors found no correlation between the overshoot magnitude of the studied parameters and the LVEF whereas significant correlations were found between the overshoot magnitude and several CPET indices of cardiorespiratory fitness, suggesting a relationship between the recovery metrics and the cardiopulmonary function during exercise but not with the resting cardiac function [12].

Considering the possible impact of peripheral (e.g., muscular and vascular) factors in the genesis and modulation of this phenomenon, the behavior of these respiratory gas indices during recovery should also be studied in a population of patients with peripheral limitations to exercise in the absence of major cardiac limitations [3,13,14]. In this context, kidney transplant recipients (KTRs) are patients with a generally reduced cardiovascular fitness also due to long-term uremia. Indeed, muscular and microvascular alterations have been reported in patients with end-stage renal disease, showing a reduced capillary density, a reduced mitochondrial density and/or function and an increased diffusion distance [15,16].

The aim of the present study was thus to characterize the recovery of the respiratory gas indices in a population of patients with peripheral limitations to exercise in the absence of significant heart disease [13,14]. The RER was chosen as the most representative metric to study as it reflects the behavior of both VO_2_ and VCO_2_.

## 2. Materials and Methods

This was a retrospective cross-sectional study that included all KTRs who underwent a functional evaluation at the Sports and Exercise Medicine Division of the Padova University Hospital between 2015 and 2018 for a cardiovascular screening, an individual assessment of physical fitness and/or an exercise prescription.

The exclusion criteria for exercise testing were an abuse of psychotropic substances, orthopedic problems that contraindicated the performance of an exercise test, a recent myocardial infarction, a known aneurysm of the thoracic aorta, recent (less than 2 months) thoracic and/or abdominal surgery and a recent pneumothorax. Patients with less than two minutes of monitored gas exchange during recovery after the test were also excluded. All subjects underwent a functional evaluation after signing their informed consent. The presented data are secondary study outcomes obtained from an action research project that derive from a diagnostic-therapeutic pathway of clinical assistance routinely performed for these patients and approved by the local Ethics Committee (protocol n. 4777/AO/19).

### 2.1. Exercise Testing Protocol

For each patient, medical and drug histories were taken, a physical examination was performed and the most recent blood tests were used to determine blood hemoglobin, glycemia, total cholesterol, high-density lipoprotein-cholesterol, low-density lipoprotein-cholesterol, triglycerides and creatinine concentration. Exercise testing was performed on a cycle-ergometer (Cycle-ergometer eBike, General Electrics) or a treadmill (COSMOS model T170 DE-med) based on the preferences or comorbidities of the patients [1].

The ramp test protocol was adapted based on the characteristics of each patient and their reported level of physical activity with the aim of reaching exhaustion within 8–12 min of incremental exercise. This maximal CPET was performed until patients reached a Borg rating of perceived exertion (RPE) ≥ 18/20. Continuous monitoring of the electrocardiogram was performed throughout the test and blood pressure was measured both during exercise and at recovery. The respiratory gas exchange (VO_2_, VCO_2_) and ventilation (VE) were monitored breath by breath during the whole test and the first 2–6 min of recovery (Masterscreen CPX system Jaeger, Carefusion, Hoechberg, GE). The peak VO_2_ was defined as the highest value of VO_2_ attained in a 30 s interval at peak exercise. The age-predicted heart rate (HR) was calculated as: (220-age) bpm. The ventilatory threshold (VT) was identified using the simplified V-slope method; in case the VT was not clearly detectable with this method, it was evaluated by two expert physicians considering the behavior of ventilatory equivalents. The respiratory compensation point (RCP) was also evaluated by two expert physicians considering the behavior of ventilatory equivalents and PETCO_2_. The VE/VCO_2_ slope was calculated as the coefficient of the linear regression obtained by plotting the VE and VCO_2_ data from the beginning of the exercise to the RCP. For patients who performed cycle-ergometer testing, the VO_2_/Watt slope was calculated as the linear regression coefficient of the relationship between VO_2_ and workload of the entire exercise phase. The oxygen uptake efficiency slope (OUES) was calculated as the coefficient of the linear relationship between oxygen uptake and the logarithm of total ventilation [17].

### 2.2. Overshoot Analyses

The behavior of the RER during recovery was analyzed and assessed four parameters: the RER at peak exercise (peak RER), the maximum RER value reached during recovery (RER max), the magnitude of the RER overshoot (RER mag) and the linear slope of the RER increase after the end of exercise.

**Figure 1:**
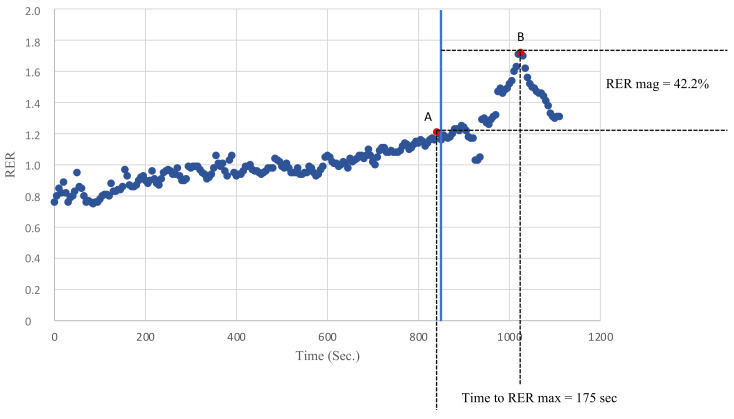
An example of the recovery of the RER in one of the KTRs. Point A defines the peak RER as the highest RER value recorded during exercise. Point B defines the RER max as the highest RER value recorded during recovery. The time to the RER max was defined as the time needed to go from A to B (s). The RER mag was defined as the percentage increase in the RER during recovery compared with the peak RER.

Breath by breath data were analyzed on an average of 5 s to avoid possible confounding values due to inconstant ventilation or sampling errors. The peak RER was defined as the highest value of the RER reached during exercise and the RER max was defined as the highest value of the RER reached during recovery. The RER slope was calculated by linearly regressing the RER data points against the time from the peak RER to the RER max. Finally, the magnitude of the RER overshoot was calculated as the percentage increase in the RER during recovery compared with the peak RER, as previously described [12]. Figure 1 describes how the peak RER, RER max, RER mag and the time to RER max were evaluated.

### 2.3. Subgroup Analysis

Two subpopulations of KTRs were further derived from the initial KTR cohort in order to investigate the behavior of the RER mag in the subgroups with potential functional and prognostic differences. Firstly, patients were divided based on the VE/VCO_2_ slope into ventilatory class I (VE/VCO_2_ slope < 30) and II (VE/VCO_2_ slope between 30 and 35.9). In addition, patients in Weber’s class A (peak VO_2_ > 20 mL/kg/min) were compared with their counterparts in classes B and C (peak VO_2_ between 16–19.9 mL/kg/min and 10–15.9 mL/kg/min, respectively).

### 2.4. Statistical Analyses

The normality was assessed using the Shapiro–Wilk test. Data are expressed as a mean ± the standard deviation. The difference between the subgroups was assessed with a *t*-test for the normally distributed variables and a Mann–Whitney U test for the non-normally distributed variables. The correlations were evaluated with Pearson’s correlation index if they were normally distributed and Spearman’s correlation index if they were non-normally distributed. A standard multiple regression was performed to assess the possible determinants of the RER mag. A statistical analysis was performed using IBM SPSS Statics software version 25. A statistical significance level of *p* ≤ 0.05 was used for all analyses.

## 3. Results

A total of 79 patients were included in the study at first. Of these, 9 were excluded because a clear overshoot could not be defined in the recorded recovery interval; i.e., the RER descent after the maximum RER reached during recovery (RER max) was not observed. Of the remaining subjects, 2 patients with a documentation of an impaired LVEF in their medical history were excluded to avoid possible confounding effects due to cardiac limitations. Finally, 11 patients with submaximal tests (e.g., a RER < 1.10 or an early interruption of exercise testing due to reasons other than exhaustion) were also excluded. The study population therefore consisted of 57 KTRs; 40 performed exercise testing on a treadmill and 17 on a cycle-ergometer. The characteristics of the patients are described in Table 1.

This sample included 16 patients (28.1%) with a history of chronic glomerulonephritis, 11 (19.3%) with polycystic kidney disease (PKD), 8 (14.0%) with diabetic nephropathy, 5 (8.8%) with a congenital malformation or renal genetic syndrome, 2 (3.5%) with interstitial nephritis, 2 (3.5%) with a combination of more than one disease and 1 (1.8%) patient for each of the following categories: nephroangiosclerosis, vasculitis, lupus nephropathy, focal segmental glomerulosclerosis, neoplasia, Alport’s syndrome and nephrotoxicity. In 6 patients (10.5%), the etiology of the renal disease was not known.

Patients exercised until perceived exhaustion (rate of perceived exertion ≥18/20 of the Borg Scale) without reporting any adverse events. The results of the cardiopulmonary exercise testing are described in Table 2.

The average time interval of the recorded recovery was 205.9 ± 56.3 s. All included patients displayed an overshoot of the RER after exercise. Patients showed a RER max of 1.60 ± 0.18 within, on average, 132.9 ± 44.7 s of recovery. The magnitude of the RER overshoot was 28.0 ± 4.1% and the RER recovery slope was 19.8 ± 14.6 (Table 2).

Correlations were assessed between the recovery metrics under study and the other common CPET parameters. Although the time to reach the RER max and the linear slope of the RER at recovery showed few and weak correlations with the main CPET parameters, the RER max and RER mag were found to be significantly correlated with most of the indices of cardiorespiratory fitness and efficiency, i.e., peak VO_2_, VO_2_ at the VT, the OUES and the VE/VCO_2_ slope (Table 3).

**Figure 2:**
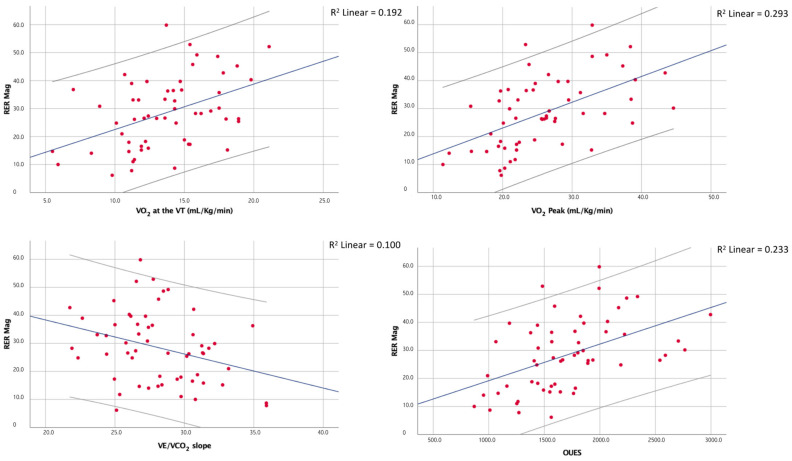
Correlations between the magnitude of the overshoot of the RER and a few of the main CPET indices of cardiorespiratory fitness and efficiency.

Moreover, although the RER max correlated with the peak RER (ρ = 0.50; *p* < 0.01), the latter showed no significant correlation with the RER mag. The main correlations of the RER mag are shown in Figure 2.

At the multiple linear regression, the peak VO_2_ was the only determinant of the RER mag between the chosen regressors even though the overall fit of the model was relatively poor (adjusted R^2^ = 0.25, *p* < 0.01). The results of the multiple regression analyses are shown in Table 4.

**Figure 3:**
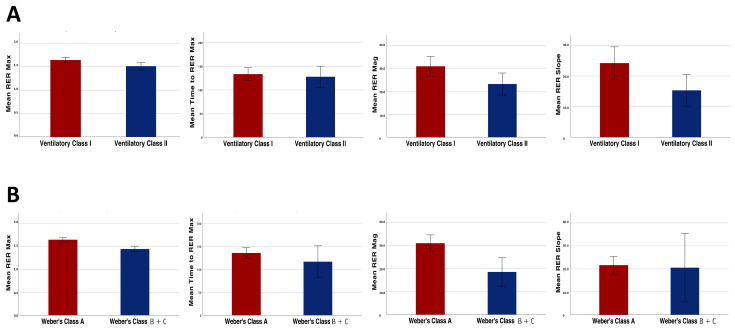
Differences in the recovery parameters (RER max, RER mag, RER recovery slope and time to reach RER max) between the patients belonging to different ventilatory efficiency classes (**A**) and Weber’s fitness classes (**B**).

With regard to the analyses performed in the KTR subgroups, the RER mag as well as the other overshoot parameters were significantly higher in the patients of ventilatory class I compared with those of ventilatory class II (30.9 ± 13.3% vs. 23.2 ± 9.6%, *p* = 0.03; Figure 3, panel A).

Similar results were found when dividing the patients based on their aerobic capacity. In fact, the RER mag was significantly higher in the patients of Weber’s class A when compared with the patients of Weber’s class B and C (31.1 ± 12.1% vs. 18.6 ± 9.9%, *p* < 0.01; Figure 3, panel B).

Finally, in our population of KTRs, 26 patients (46%) were taking beta-blockers at the time of the CPET evaluation. A comparison between these patients and those not taking beta-blockers showed that, despite an expected lower peak HR (77.9% of the age-predicted, *p* < 0.01) and a few significant differences in peak VO_2_ (23.4 ± 6.4 vs. 27.9 ± 6.8 mL/kg/min, *p* = 0.02) and VO_2_ at the VT (12.6 ± 3.3 vs. 14.5 ± 3.3 mL/kg/min, *p* = 0.04), the RER mag was not significantly different between the two subpopulations (27.1 ± 14.6% vs. 29.6 ± 11.0%, *p* = 0.47), nor were the other recovery metrics.

## 4. Discussion

To the best of the authors’ knowledge, this is the first study that has evaluated the overshoot parameters of the respiratory gas exchange and in particular the behavior of the RER during recovery from maximal exercise testing in KTRs. Among the variables studied, the magnitude of the RER overshoot appeared to be the most meaningful as it was significantly correlated with the other indices of cardiorespiratory fitness and efficiency, independent from the peak RER. This study thus provides new and useful reference values of these parameters for a population of KTRs. It was also shown how the phenomenon of the RER overshoot seems to be tightly correlated with the cardiopulmonary function, resulting in a reduction in patients with clinically impaired functional capacity and cardiopulmonary efficiency, two known prognostic CPET markers. Finally, beta-blockers were shown to have no impact on the RER behavior during recovery in our cohort.

To date, compared with the extended body of literature dedicated to the cardiopulmonary response to exercise in different clinical populations, there is relatively less evidence on the behavior of CPET indices during recovery, particularly regarding their clinical and pathophysiological significance. The recovery kinetics of VO_2_ have been related to the O_2_ debt in healthy subjects [5]. Previous studies have demonstrated how, during submaximal exercise, the recovery kinetics of VO_2_ are more reproducible than the VO_2_ onset kinetics and how they can help to discriminate patients with heart failure from their healthy counterparts [18]. Other authors have described a delay in the recovery of VO_2_ after maximal and submaximal incremental exercise testing in patients with heart failure compared with normal subjects, which was correlated with the disease severity [5,6]. In these works, the VO_2_ recovery delay was mainly attributed to a slow recovery of the energy stores of the muscles, independent from the achieved workload at peak exercise. Moreover, Cohen-Solal et al. also found a delay in the recovery of VCO_2_ and VE in cardiac patients similar to that of VO_2_. The explanation for this finding was attributed to a retention of CO_2_ in the exercising muscles, which also justified the excessive ventilation needed to maintain eucapnia [5]. In light of these findings, it is interesting to observe how the recovery parameters seemed to better correlate than the peak parameters with muscle strength in both patients with heart failure and healthy controls [19].

Another associated phenomenon that has been described for patients with heart failure is the overshoot of VO_2_ after exercise interruption. This is defined as a further increase in VO_2_ during recovery compared with the values observed at peak exercise [9,10], which has been found with a variable prevalence among cardiac patients and also in association with a worse prognosis [20]. A concomitant overshoot of the cardiac output in patients with heart failure has been described by other authors, suggesting that the VO_2_ overshoot could be explained by a paradoxical increase in the stroke volume, probably due to a decrease in the peripheral vascular resistances after stopping exercise [9,11]. The authors also hypothesized that the increased stroke volume could be distributed to skeletal muscles to repay the oxygen deficit or that it could depend on a relatively slow decline of catecholamines at the end of exercise.

More recently, Takayanagi et al. focused on the overshoot of several other CPET indices, i.e., the RER, PETO_2_ and VE/VO_2_. It was demonstrated how an overshoot of these variables is commonly seen in the recovery phase after maximal exercise testing and how this overshoot is attenuated in magnitude among cardiac patients with a LVEF < 40% when compared with age-matched healthy subjects [12]. The authors attributed this impairment to the delayed recovery of VO_2_ previously described in patients with heart disease. Moreover, they found a good correlation of the overshoot magnitude with cardiorespiratory fitness and peak work rate but no correlation with the LVEF, suggesting a tight relationship of these metrics with the cardiopulmonary function during exercise but not with the cardiac function at rest.

KTRs are patients who usually carry several complications from chronic kidney disease leading to a transplantation. Anemia, autonomic dysfunction and cardiac and/or vascular dysfunction are common conditions in end-stage renal disease [16]. In addition, alterations at the muscular and microvascular level have been reported in this population, showing a reduced capillary density, a reduced mitochondrial density and/or function and an increased diffusion distance [15,16]. As the current literature is primarily focused on the cardiopulmonary response during recovery from exercise in patients with major cardiac limitations, it was the aim of the present study to assess the recovery behavior of the respiratory gas exchanges in a population without major cardiac limitations but probable peripheral limitations to exercise. In this context, the RER was chosen as the most representative metric to study as it reflects the behavior of both VO_2_ and VCO_2_.

The present study confirmed the occurrence of an overshoot of the RER during recovery from maximal exercise testing in all (100%) evaluated patients and it was the first to provide values of the RER mag, RER max, time to reach RER max and RER recovery slope in a population of KTRs. Most of these metrics are poorly described in the literature but could help in understanding the recovery kinetics of VO_2_ and VCO_2_. Given the retrospective nature of this study, the overshoot of the RER was not assessed for a control group, thus limiting further considerations. However, comparing our results with previous study outcomes, the magnitude of the RER overshoot of KTRs (28.4 ± 12.7%) was comparable with that found in healthy subjects (29.3 ± 10%) [12]. This finding suggests that peripheral alterations, such as physical deconditioning and muscular and microvascular abnormalities that are typically present in KTRs [15,16], are not sufficient to determine an attenuation of this physiological phenomenon and that cardiac limitations to exercise could have a major impact on the magnitude of the RER overshoot.

Additionally, the analysis of the RER during recovery in this population provided interesting findings, showing that this parameter correlates with physical fitness and cardiorespiratory efficiency even in KTRs, thus suggesting its possible integration in the interpretation of CPET in clinical populations. The RER overshoot showed significant correlations with other important indices of cardiopulmonary functions such as peak VO_2_, VO_2_ at the first VT, the OUES and the VE/VCO_2_ slope. The correlations with the VO_2_/WR slope, found in a previous study on the RER overshoot [12], might have been reduced significantly in the present study due to the low number of subjects that performed exercise testing on a cycle-ergometer. Among all the metrics evaluated in the present study, the RER max and RER mag displayed the strongest correlations with other cardiopulmonary function indices. Moreover, the correlations of the RER mag with the peak VO_2_ (ρ = 0.571, *p* < 0.01), VO_2_ at the VT (r = 0.438, *p* < 0.01) and the VE/VCO_2_ slope (r = −0.316, *p* < 0.05) were comparable with those previously described for cardiac patients (r = 0.50, r = 0.42 and r = −0.36, respectively; all *p* < 0.05) [12]. This fact consolidates the validity of this metric in a cohort of patients affected by a chronic disease without systolic dysfunction, opening perspectives to its evaluation even in other clinical settings. Moreover, the RER mag appears to be a more independent and thus useful parameter as it was found to be independent from the peak RER. Multiple regression analyses provided further confirmation that the RER overshoot was strongly influenced by the level of cardiovascular fitness and not by other determinants such as VCO_2_, hemoglobin and age. However, it stands to reason that future studies, including other clinical variables related to the study subjects in addition to the cardiorespiratory function parameters, may help to explain other aspects of this phenomenon.

**Figure 4:**
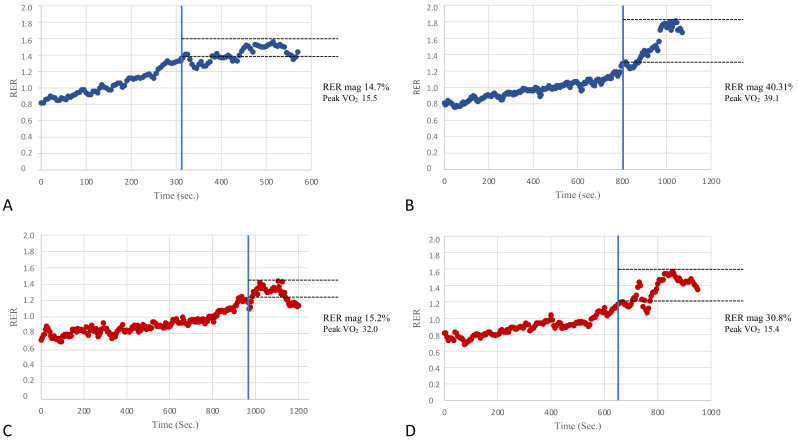
A few visual examples of the RER mag. Panels (**A**,**B**) show two examples of poor (14.7%) and good (40.31%) RER mag in patients of our population with relatively poor and good cardiovascular fitness as per the peak VO_2_ (Weber’s class C and A, respectively). On the other hand, panels (**C**,**D**) show two examples of a poor (15.2%) and good (30.8%) RER mag in patients with relatively good and poor cardiovascular fitness (Weber’s class A and C, respectively). The RER mag appears to be significantly correlated with cardiorespiratory fitness in the present study; however, this figure suggests that it might also be useful to add the RER overshoot information in the CPET evaluation of patients even in the presence of a normal aerobic capacity and vice-versa.

In an attempt to assess the potential clinical value of the RER overshoot, KTRs were divided into subgroups based on two classifications used in the prognostic risk stratification of patients with heart failure [4]. Moreover, patients were divided by Weber’s class into those with better fitness belonging to Weber’s class A (peak VO_2_ > 20 mL/kg/min) and patients belonging to classes B and C (peak VO_2_ between 16–19.9 mL/kg/min and 10–15.9 mL/kg/min, respectively). Patients with low fitness levels were pooled due to the small sample size. The inter-group comparisons showed that the RER mag was significantly different (31.1 ± 12.1% vs. 18.6 ± 9.9%, respectively, *p* < 0.01). In addition, the patients were also divided according to their ventilatory classes, which reflected the respiratory efficiency and degree of the ventilation-perfusion mismatch during exercise. Patients belonging to ventilatory class I (VE/VCO_2_ slope < 30) showed significant differences compared with those belonging to ventilatory class II (VE/VCO_2_ slope between 30 and 35.9) in peak VO_2_ and also in the RER mag (30.9 ± 13.3% vs. 23.2 ± 9.6%, *p* = 0.03). Therefore, the RER mag values were altered in those patients belonging to the worse prognostic classes (i.e., Weber’s class B + C and ventilatory class II), in line with what has been previously reported in cardiac patients with a reduced LVEF [12]. Figure 4 provides a few visual patterns of the RER mag among patients with normal or impaired cardiorespiratory fitness.

Looking at these data, it might be tempting to speculate that not only VO_2_ but also VCO_2_ plays a role in reducing the RER overshoot, possibly through CO_2_ retention within exercising muscles, therefore showing higher VE/VCO_2_ slopes during exercise. However, our data impose caution with this consideration as peak VCO_2_ was not a determinant of the RER mag in the multiple regression analyses. It is possible that the spread of a ventilation-perfusion mismatch in this population was too limited to give strength to the correlation between the RER mag and peak VCO_2_ at the multiple regression. Indeed, no patients were found in ventilatory classes > II. In the perspective of future studies, it could therefore be specifically investigated what causes the RER to move in the recovery phase of exercise testing in healthy and diseased patients.

So far, the recovery phase after exercise has been studied in relatively few clinical populations and often by applying different methods and metrics. The present data seem promising in the perspective of integrating a CPET evaluation with different variables of recovery, which might help to improve the risk stratification of these patients. The RER mag appeared to be the most useful and feasible metric that can also be evaluated in the clinical setting (Figure 4). Further studies are needed to evaluate the reproducibility of this metric and to assess its pathophysiological determinants.

### Limitations and Perspectives

This was a retrospective trial evaluating the recovery phase after maximal exercise by CPET evaluations performed for clinical purposes. There was, therefore, a lack of standardization in the protocol used and in the monitored gas exchange recovery time interval, which could have led to a possible underestimation of the RER overshoots for patients with a shorter monitored recovery. Indeed, future specifically designed experimental studies should investigate how exercise modality, test protocols and training interventions might affect these overshoot parameters [21]. Furthermore, an evaluation of the RER recovery could not be provided for a matched control population, limiting the qualitative considerations of the metrics studied. The medical history and biochemical parameters of the study subjects were also assessed retrospectively, based on clinical records, and were thus not standardized in terms of clinical evaluations and the time distance of the laboratory tests from CPET. The classifications used to divide patients in prognostic CPET classes were designed for patients with heart failure and might not be optimal to clinically stratify KTRs; however, given the absence of reference values for this population, they were the only criteria suitable for this purpose. Finally, the time from the transplant of our cohort of KTRs was variable and it was not possible to exclude that patients with older transplants could have improved their muscular and microvascular function compared with patients with a more recent transplantation. However, future trials should assess the recovery of the respiratory gas exchange after physical exercise in populations with different functional limitations in order to provide more evidence with regard to the underlying pathophysiological mechanisms of the overshoot and its clinical interpretation.

## 5. Conclusions

The present study adds new evidence to a promising issue for a functional evaluation in patients with chronic diseases. The recovery of the respiratory gas exchange parameters was studied in KTRs, evaluating the RER overshoot after maximal exercise testing. Our data showed the occurrence of a RER overshoot during recovery in all evaluated KTRs with a magnitude of 28.4 ± 12.7%, which is comparable with that of healthy subjects previously reported in the literature. A significant correlation of the RER overshoot with the CPET indices of cardiorespiratory fitness and efficiency was confirmed. Moreover, the magnitude of the RER overshoot was lower in patients with decreased cardiovascular fitness and in those with a significant ventilation-perfusion mismatch but it was not affected by beta-blocker therapy. Finally, the magnitude of the RER overshoot appeared the most meaningful metric to assess the recovery of patients, being independent from the peak RER. Thus, the evaluation of the RER recovery should be included in clinical CPET interpretations to provide more scientific data and further improve prognostic risk stratification in KTRs and other chronic diseases.

## Figures and Tables

**Table 1:** Clinical characteristics of the study population (*n* = 57) of kidney transplant recipients.

Female/Male	16/41
Age (years)	51.4 ± 13.0
Height (cm)	170.6 ± 8.4
Weight (kg)	70.4 ± 10.8
BMI (kg/m^2^)	23.3 ± 3.1
Hb (g/L)	126.4 ± 16.4
Glycemia (mmol/L)	5.7 ± 1.6
Blood cholesterol (mmol/L)	5.5 ± 1.2
HDL-cholesterol (mmol/L)	1.4 ± 0.5
LDL-cholesterol (mmol/L)	3.3 ± 1.0
Triglycerides (mmol/L)	1.8 ± 0.8
Blood creatinine concentration (μmol/L)	132.7 ± 35.0

Data are expressed as a mean ± the standard deviation. BMI = body mass index; Hb = hemoglobin concentration.

**Table 2:** Cardiopulmonary test parameters and recovery metrics of the whole population of kidney transplant recipients.

Exercise Parameters	Mean ± SD
Peak HR (bpm)	146.0 ± 22.5
Peak HR (% of predicted)	86.7 ± 12.4
VO_2_ at the VT (mL/min)	950.8 ± 251.6
VO_2_ at the VT (mL/kg/min)	13.6 ± 3.4
Peak VO_2_ (mL/min)	1805.1 ± 549.2
Peak VO_2_ (mL/kg/min)	25.9 ± 7.5
Peak VO_2_ (% of predicted)	87.4 ± 21.2
Peak VCO_2_ (mL/min)	2179.9 ± 714.1
VE/VCO_2_ slope	28.2 ± 3.3
OUES (mL/log·L)	1708.2 ± 469.6
HR/VO_2_ slope	4.3 ± 1.3
VO_2_/work rate slope (mL/Watt)	9.6 ± 1.9
Recovery Parameters	Mean ± SD
Peak RER	1.25 ± 0.08
RER max	1.60 ± 0.18
Time to reach RER max (s)	131.4 ± 42.8
Magnitude of the RER overshoot (%)	28.4 ± 12.7
RER recovery slope	21.4 ± 15.3

Data are expressed as a mean ± the standard deviation. HR = heart rate; VO_2_ = oxygen uptake; VT = ventilatory threshold; VE/VCO_2_ slope = minute ventilation/carbon dioxide production slope; VCO_2_ = carbon dioxide production; OUES = oxygen uptake efficiency slope; RER = respiratory exchange ratio.

**Table 3:** Correlations between the RER recovery and CPET parameters expressed as Pearson’s r for the normally distributed data and Spearman’s ρ for the non-normally distributed data.

	Peak RER	RER Max	RER Mag	RER Slope	Time to RER Max
Age	-	−0.325 *	−0.266 *	-	-
Peak HR (bpm)	-	0.295 *	0.302 **	-	-
HR/VO_2_ slope	-	-	-	-	-
Hb (g/L)	-	-	0.264 *	0.309 *	-
VO_2_ @ VT (mL/kg)	-	0.295 *	0.438 **	-	-
Peak VO_2_ (mL/kg)	-	0.597 **	0.571 **	-	-
Peak VO_2_ (% of predicted)	-	-	-	-	-
Peak VCO_2_ (mL/min)	-	0.566 **	0.495 **	-	-
VE/VCO_2_ slope	−0.392 **	−0.512 **	−0.316 *	−0.298 *	-
VO_2_/WR slope (mL/Watt)	-	-	-	-	-
OUES (mL/log L)	-	0.380 **	0.483 **	-	-

* Correlation significant at the 0.05 level. ** Correlation significant at the 0.01 level. HR = heart rate; VO_2_ = oxygen uptake; VT = ventilatory threshold; VCO_2_ = carbon dioxide production; VE/VCO_2_ slope = minute ventilation/carbon dioxide production slope; OUES = oxygen uptake efficiency slope; RER = respiratory exchange ratio.

**Table 4:** Results of the multiple linear regression.

			95% Confidence Interval	
	Standardized Beta	*p*	Lower Bound	Upper Bound
VO_2_ (mL/kg/min)	0.54	0.02	0.14	1.7
Peak VCO_2_ (mL/Watt)	−0.08	0.74	−0.01	0.01
Age	−0.08	0.54	−0.33	0.18
Hb (g/L)	0.11	0.44	−0.13	0.29

VO_2_ = oxygen uptake; VCO_2_ = carbon dioxide production; Hb = hemoglobin concentration.

## Data Availability

The datasets generated during and/or analyzed during the current study are not publicly available but are available from the corresponding author on reasonable request.
